# Current practices in ICU delirium management: a prospective multicenter study in the Netherlands

**DOI:** 10.1186/cc12333

**Published:** 2013-03-19

**Authors:** Z Trogrlic, E Ista, A Slooter, J Bakker, M Van der Jagt

**Affiliations:** 1 Erasmus MC University Medical Center, Rotterdam, the Netherlands

## Introduction

As part of a multicenter prospective study on barriers and facilitators for implementation of protocolled care for delirious critically ill patients, we aimed to describe current practices in delirium management in a representative cohort of ICU patients.

## Methods

We included consecutively admitted patients during a 4-month period in a prospective multicenter study in six ICUs (one academic, teaching and nonteaching hospitals) in the southwest of the Netherlands. We assessed: percentage of patients screened for delirium with validated screening tools; pharmacological treatment with haloperidol or other antipsychotic drugs; psychohygiene (use of hearing aids and or glasses, preventing sleep disturbances); and access to early mobilization and physiotherapy. Delirium was pragmatically defined as administration of haloperidol and/or delirium reported by physicians or ICU nurses in patient records, as assessed by a designated research nurse. Differences between centers were tested with non-parametric tests.

## Results

We assessed 1,576 patients, corresponding with 8,150 ICU treatment days with a median length of stay of 3 days (IQR 2 to 5). The mean age of the patients was 62 years (SD = 16) and 58% were male. Delirium occurred in 23% (356/1,576) of patients with a median duration of 3 days (IQR 2 to 7) and ranged from 11 to 40% for each ICU. Delirium assessment with the CAM-ICU at any point during ICU stay was performed in 38% of all patients. Screening with CAM-ICU was applied in three ICUs, in 29 to 96% of the patients in these centers. Of 3,564 documented screening days with the CAM-ICU, it was positive in 1,459 (41%); in only 120 (8%) of these CAM-ICU-positive days there was a documented action or treatment started for delirium. However, patients still received haloperidol on 52% (*n = *766) of all CAM-ICU-positive days. Patients received benzodiazepines in 49% (*n = *1,141) of patient sedation days. Delirium preventive interventions were physiotherapy (19% of 8,150 ICU days), mobilization (10%), glasses use (2.6%) and hearing aid use (0.3%). Presence of hearing or visual impairment at admission was not documented in 65% of patients.

## Conclusion

Daily screening for ICU delirium with a validated screening instrument is applied in less than one-half of the time in critically ill patients and management of delirium is often not guided by this screening. Haloperidol was used as the first-choice medication. Measures aimed at delirium prevention (psychohygiene and early mobilization) were carried only in a small minority or were not documented. To implement protocolled delirium care in the region at study, a multifaceted tailored implementation program is needed.

